# Pollen Number and Ribosome Gene Expression Altered in a Genome-Editing Mutant of *REDUCED POLLEN NUMBER1* Gene

**DOI:** 10.3389/fpls.2021.768584

**Published:** 2022-01-11

**Authors:** Hiroyuki Kakui, Takashi Tsuchimatsu, Misako Yamazaki, Masaomi Hatakeyama, Kentaro K. Shimizu

**Affiliations:** ^1^Department of Evolutionary Biology and Environmental Studies, University of Zurich, Zurich, Switzerland; ^2^Kihara Institute for Biological Research, Yokohama City University, Yokohama, Japan; ^3^Graduate School of Science and Technology, Niigata University, Niigata, Japan; ^4^Department of Plant and Microbial Biology & Zurich-Basel Plant Science Center, University of Zurich, Zurich, Switzerland; ^5^Gregor Mendel Institute, Austrian Academy of Sciences, Vienna BioCenter, Vienna, Austria; ^6^Department of Biology, Chiba University, Chiba, Japan; ^7^Department of Biological Sciences, University of Tokyo, Tokyo, Japan; ^8^Functional Genomics Center Zurich, Zurich, Switzerland

**Keywords:** pollen number, *REDUCED POLLEN NUMBER1*, complementation test, CRISPR/Cas9, transcriptome, ribosomal protein

## Abstract

The number of pollen grains varies within and between species. However, little is known about the molecular basis of this quantitative trait, in contrast with the many studies available on cell differentiation in the stamen. Recently, the first gene responsible for pollen number variation, *REDUCED POLLEN NUMBER1* (*RDP1*), was isolated by genome-wide association studies of *Arabidopsis thaliana* and exhibited the signature of natural selection. This gene encodes a homolog of yeast Mrt4 (mRNA turnover4), which is an assembly factor of the large ribosomal subunit. However, no further data were available to link ribosome function to pollen development. Here, we characterized the *RDP1* gene using the standard *A. thaliana* accession Col-0. The frameshift mutant, *rdp1-3* generated by CRISPR/Cas9 revealed the pleiotropic effect of *RDP1* in flowering, thus demonstrating that this gene is required for a broad range of processes other than pollen development. We found that the natural Col-0 allele conferred a reduced pollen number against the Bor-4 allele, as assessed using the quantitative complementation test, which is more sensitive than transgenic experiments. Together with a historical recombination event in Col-0, which was identified by sequence alignment, these results suggest that the coding sequence of *RDP1* is the candidate region responsible for the natural phenotypic variation. To elucidate the biological processes in which *RDP1* is involved, we conducted a transcriptome analysis. We found that genes responsible for ribosomal large subunit assembly/biogenesis were enriched among the differentially regulated genes, which supported the hypothesis that ribosome biogenesis is disturbed in the *rdp1-3* mutant. Among the pollen-development genes, three key genes encoding basic helix-loop-helix (bHLH) transcription factors (*ABORTED MICROSPORES* (*AMS*), *bHLH010*, and *bHLH089*), as well as direct downstream genes of *AMS*, were downregulated in the *rdp1-3* mutant. In summary, our results suggest a specialized function of ribosomes in pollen development through *RDP1*, which harbors natural variants under selection.

## Introduction

Pollen grain number in seed plants is a key reproductive trait that has been studied extensively for decades, from agricultural and evolutionary viewpoints. Domesticated crop species generally produce fewer pollen grains than wild relative species, whereas a high pollen number is preferred in some cultivation strategies, such as hybrid crops and artificial pollination (Oka and Morishima, 1967; Franco-Mora et al., 2005; Langer et al., 2014; Shimizu and Tsuchimatsu, 2015; Boeven et al., 2016). Despite its agricultural and evolutionary importance, the genetic basis of pollen grain number has remained elusive, mainly because of its quantitative nature. Recently, the *REDUCED POLLEN NUMBER1* (*RDP1*) gene, which is responsible for the natural variation in pollen number, was identified through a genome-wide association study of *Arabidopsis thaliana* (Tsuchimatsu et al., 2020). The CRISPR/Cas9-generated *RDP1* frameshift mutants in the Col-0 background produced about half of the number of pollen grains compared with wild-type and was considered non-functional or null. The Uod-1 accession had a long-haplotype variant at the *RDP1* region, which conferred a lower number of pollen grains and exhibited a signature of selective sweep. The Bor-4 accession had an alternative variant that conferred a greater number of pollen grains. Both variants did not contain a gene disruptive mutation. Causal evidence of the allelic effects of *RDP1* was obtained using Uod-1 and Bor-4 via the quantitative complementation test (also known as reciprocal hemizygosity test) (Tsuchimatsu et al., 2020), which compares allelic effects under the same copy number and positions of genes (Stern, 1998, 2014; Turner, 2014). Although functional tests using transgenesis are often used to examine exact causal mutations, a subtle allelic effect may not be detected in this manner because of the uncertainty of the insertion position in the genome in plants. Further quantitative complementation testing using an accession that experienced historical recombination events (such as Col-0) is another experiment that can narrow down the specific region responsible for the allelic effect of *RDP1*.

In contrast with pollen-number regulation, the molecular basis of cell differentiation in stamen development has been well studied (Alvarez-Buylla et al., 2010; Walbot and Egger, 2016; Ferguson et al., 2017; Li et al., 2017; Xue et al., 2021). Pollen and tapetal cell lineages differentiate from archesporial cells, with the latter being essential for the development of the former (Sanders et al., 1999; Feng and Dickinson, 2010; Walbot and Egger, 2016). The development of tapetum and pollen lineages is governed by a genetic pathway called “DYT1-TDF1-AMS-MS188-MS1” (Li et al., 2017), which is composed of transcriptional factors (Zhu et al., 2011; Lou et al., 2018). Two of them, DYSFUNCTIONAL TAPETUM1 (DYT1) and ABORTED MICORSPORES (AMS), are basic helix-loop-helix (bHLH) transcription factors that interact with three other bHLH factors, bHLH10, bHLH89, and bHLH91. Their protein interactions and transcriptional feedback regulations were reported (Feng et al., 2012; Zhu et al., 2015; Cui et al., 2016). Among these bHLH factors, AMS is known as a master regulator of pollen wall formation (Sorensen et al., 2003; Xu et al., 2010; Zhu et al., 2011; Xu et al., 2014). Transcriptome data showed that many genes are differentially expressed between wild-type and *ams* mutant. Li et al. (2017) detected 825 downregulated genes from tapetum cells in the *ams* mutant. Xu et al. (2014) reported 23 genes that were directly regulated by AMS based on microarray and qChIP-PCR experiments. Based on these detailed characterizations of transcriptional regulation and protein interaction in the pollen/anther pathway (Zhu et al., 2011; Ma et al., 2012; Cui et al., 2016; Ferguson et al., 2017; Wang et al., 2018; Chen et al., 2019), we investigated these pollen development genes in the mutants of *RDP1*.

*RDP1* encodes a protein with homology to the mRNA turnover 4 (Mrt4) protein. Mrt4 acts as a pre-60S ribosomal component in yeast (Schmidt et al., 2013; Salih et al., 2020; Tsuchimatsu et al., 2020). The *Mrt4* gene is not essential in yeast, but null mutants of *mrt4* show slow-growth phenotypes, suggesting its role in cell proliferation (Rodriguez-Mateos et al., 2009a,b). However, it is unclear whether RDP1 is working as a component of ribosome and affect the translation in *A. thaliana*. Ribosomal genes were traditionally considered housekeeping genes of the protein synthesis machinery, and to function uniformly in all cells. Nevertheless, increasing evidence has shown ribosome specialization in plant and other model species (Martinez-Seidel et al., 2020; Norris et al., 2021). Many mutants of ribosome-related genes exhibit organ specific phenotypes, such as the reduction of leaf cell number, larger leaf cell size (Fujikura et al., 2009; Horiguchi et al., 2011), reduced root length (Creff et al., 2010), and reduction of stamen number (Stirnberg et al., 2012). Transcriptome studies of ribosome biogenesis factor mutants of *Arabidopsis* reported the upregulation of ribosomal genes as well as altered regulation of other genes, which supported the importance of transcriptome data of ribosome biogenesis factors mutant (Beine-Golovchuk et al., 2018; Cheong et al., 2021).

Here, we focused on the standard Col-0 background for further characterization of the *RDP1* gene. First, the phenotypes at different developmental stages were examined in the frameshift *rdp1-3* mutant in the Col-0 background. Second, the *RDP1* genomic sequences of Col-0, Uod-1, and Bor-4 were aligned to characterize the Col-0 sequence, and the quantitative complementation test was used to narrow down the candidate regions responsible for the natural phenotypic variation. Subsequently, we performed transcriptome analyses to detect differentially expressed genes (DEGs) between the wild-type and *rdp1-3* plants focusing on ribosome-related and pollen development genes. Finally, we assessed whether ribosome genes were upregulated, as previously observed in *Arabidopsis* ribosome mutants. Our data support the specific function of the ribosome in the developing pollen cell lineage.

## Materials and Methods

### Plant Materials and Growth Conditions

Four accessions of *A. thaliana*, Col-0 (N22625), Bor-4 (N22591), Mz-0 (N22636), and Uod-1 (N22612), as well as *A. lyrata* ssp. *lyrata* (CS22696), were used in this study. *Arabidopsis* seeds were sown on soil mixed with the insecticide ActaraG (Syngenta Agro, Switzerland) and stratified for 3–4 days at 4°C in the dark. The plants were grown under 16 h of light at 22°C and 8 h of dark at 20°C, with weekly treatments with insecticide (Kendo Gold, Syngenta Agro). The frameshift mutants of *RDP1* created by CRISPR/Cas9, i.e., *rdp1-3* (Col-0) and *rdp1-6* (Bor-4), were generated previously (Tsuchimatsu et al., 2020). The frameshift of *rdp1-3* and *rdp1-6* were identical. These frameshift mutants were genotyped by PRIMA using primers (Forward primer, 5′–TAGGCACAATGGAAAGTTAG–3′; Reverse primer, 5′–TT AACATAAAAGAACCATTGTAAG–3′) and 40-mer probe (5′–GGAGACTTTGTAGATACCAGAGTTCATCTTCTGCAGAAC G–3′) as described previously (Kakui et al., 2021). T-DNA fragments containing CRISPR/Cas9 and GFP marker were removed from each strain of *rdp1-3* and *rdp1-6* by selecting non-fluorescence seeds (Tsutsui and Higashiyama, 2017). Bolting time was determined when the main inflorescence stem reached 3 cm. Flowering time was recorded when the first flower opened. Time-lapse movie of plant growth from Col-0 and *rdp1-3* was obtained using a TLC200 Pro camera (Brinno, Taiwan).

### Histological Analysis of Anthers

For histological analysis, inflorescences were fixed with FAA (formaldehyde:acetic acid:70% ethanol = 1:1:18), dehydrated, and embedded in Technovit 7100 according to the manufacturer’s instructions (Heraeus Kulzer GmbH, Wehrheim, Germany). Five-micrometer sections were cut with a microtome (RM2145, Leica, Germany) and stained with toluidine blue before observation under a Leica microscope (DM5000, Leica) equipped with a color camera (DMC2900, Leica).

### Traditional Transgenic Complementation

The *RDP1* sequence of Col-0, including the 1,960 bp upstream region from the coding sequence to 661 bp downstream from the stop codon, was amplified using the following primers: 3795_At1g25260F (5′–TTTCTCCCCACATTTCTC–3′) and 3988_At1g25260R (5′–TATGTTATCAAAATTCATAAAATG–3′). The *RDP1* sequences from different accessions were amplified from Mz-0, Col-0, Bor-4, and *A. lyrata* by PCR (PrimeSTAR GXL, Takara Bio, Japan). These PCR products were cloned into pFAST-R01 (Shimada et al., 2010). Each construct was independently transformed into *rdp1-3* (Col-0 background) plants using the floral-dip method (Clough and Bent, 1998) with *Agrobacterium tumefaciens* (GV3101).

### Quantitative Complementation

*Arabidopsis thaliana* plants with heterozygous *RDP1* alleles from two accessions, i.e., the Col-0 accession (*RDP1/rdp1-3*, termed *RDP1*^Col^/*rdp1*^Col^ hereafter) and the Bor-4 accession (*RDP1*/*rdp1-6*, termed *RDP1*^Bor^/*rdp1*^Bor^ hereafter), were prepared (Tsuchimatsu et al., 2020). F_1_ plants were generated by crossing *RDP1*^Col^/*rdp1*^Col^ and *RDP1*^Bor^/*rdp1*^Bor^. Subsequently, F1 progenies are genotyped and analyzed.

### Pollen-Number Counting Using a Cell Counter

We sampled flower buds and counted pollen numbers using a cell counter as described previously (Kakui et al., 2020; Tsuchimatsu et al., 2020). In summary, flowers of stage 12 [unopened anther with mature pollen grains (Smyth et al., 1990)] were collected and incubated overnight at 60°C. Flowers were collected from the side stem because flowers of the main stem have significantly larger pollen numbers (Tsuchimatsu et al., 2020). 1st and 2nd flowers of flower buds were excluded from sampling because they tend to show abnormal flower shapes (Kakui et al., 2020). We collected flowers from the start of flowering until approximately 2 weeks later. Subsequently, 30 μL of 5% Tween-20 was added, and the mixture was sonicated using a sonicator (Bioruptor Plus, Diagenode, Belgium), to release the pollen grains. The pollen suspension was mixed with a pollen counting solution (CASYton, OMNI Life Science, Germany), and particles were counted on a cell counter (CASY cell counter, OMNI Life Science). Pollen-number data were statistically analyzed and plots were constructed in R (R Core Team, 2013).

### Transcriptome Analysis

Total RNA was isolated from four replicates of flower buds of the wild-type and *rdp1-3* plants using the RNeasy plant mini kit (Qiagen, Germany). The samples encompassed a wide range of developmental stages (flower stages 1–12) (Sanders et al., 1999), excluding opened flowers. Next, 75-bp single-end read sequencing was performed on a NextSeq 500 sequencer (Illumina, San Diego, CA, United States). The sample information and the number of reads are listed in Supplementary Table 1. The transcriptome data obtained were analyzed using the SUSHI framework (Hatakeyama et al., 2016). We defined DEGs based on a false discovery rate (FDR) < 0.1. All DEGs data are listed in Supplementary Table 2. The gene ontology (GO) enrichment analysis was performed using the ShinyGO v0.66 software (Ge et al., 2020).

## Results

### Natural Variation of *RDP1* Sequences and the Validation of Allelic Functional Differences Using the Quantitative Complementation Test

**FIGURE 1:**
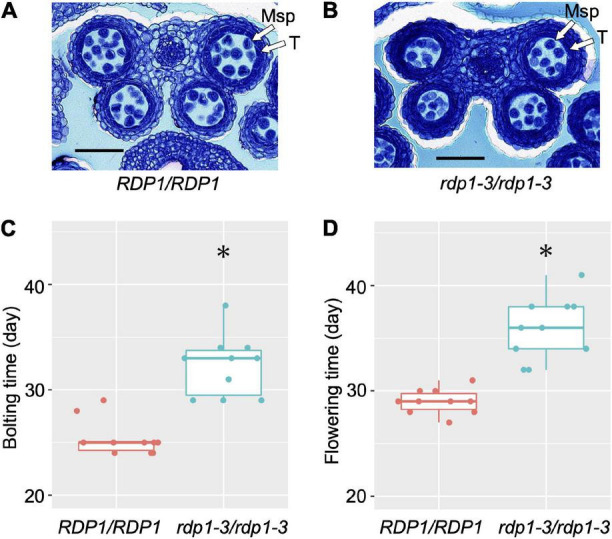
Anther sections, bolting time, and flowering time of wild-type (*RDP1/RDP1*) and null mutant (*rdp1-3/rdp1-3*) plants. Transverse sections of *RDP1/RDP1* **(A)** and *rdp1-3/rdp1-3* **(B)** anthers (anther stage 8). Msp, microspores; T, tapetum. Scale bars, 50 μm. Bolting **(C)** and flowering **(D)** time of *RDP1/RDP1* and *rdp1-3/rdp1-3* plants (*n* = 10 and 10 for **C,D**, respectively; Asterisks indicate significant differences, determined by Wilcoxon rank-sum test, *P* = 4.33e–05 for **C**, *P* = 1.08e–05 for **D**). Boxplots **(C,D)** show center line: median; box limits: upper and lower quartiles; dots: individual data points.

The CRISPR/Cas9-generated frameshift *rdp1-3* mutant in the Col-0 background produced about half the number of pollen grains compared with the wild-type counterpart (Tsuchimatsu et al., 2020). We further observed the section of the developing anther and confirmed that the number of microspores was already decreased in the mutant at the anther stage 8 (Sanders et al., 1999; Figures 1A,B). Tsuchimatsu et al. (2020) reported that the frameshift *rdp1-3* mutants exhibited other pleiotropic phenotypes, such as reduced ovule number and delayed growth, in contrast with the effect of natural variants. Here, we found that the bolting and flowering times were significantly delayed (Wilcoxon rank-sum test; *P* = 4.33e–05 for bolting time, *P* = 1.08e–05 for flowering time, Figures 1C,D and Supplementary Video 1). These results support the notion that *RDP1* functions as an Mrt4 homolog in *A. thaliana*, in accordance with the slower growth observed in the Mrt4 null mutant in yeast (Rodriguez-Mateos et al., 2009a).

**FIGURE 2:**
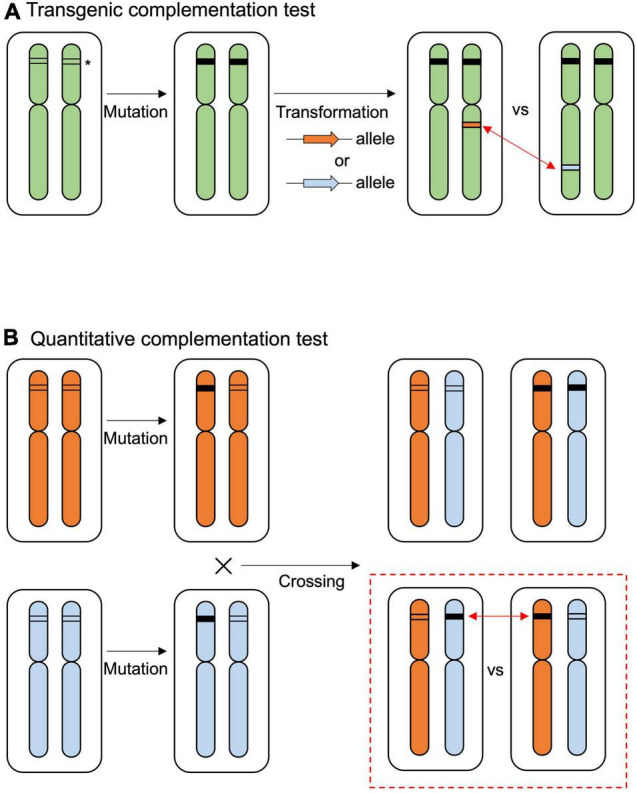
Overview of the complementation tests. **(A)** The traditional transgenic complementation test compares the effects of inserted alleles (indicated by orange or blue arrows) in the null mutant background. The allelic effect of the target gene is compared from different insertion positions (red two-sided arrows). The asterisk indicates the target gene. The black box at the target gene indicates the mutated sequence. **(B)** Quantitative complementation test using null mutation of different alleles. Allelic effects are evaluated in the F_1_ progenies of heterozygous mutants (red dashed square). The allelic effect of the target gene is compared from the original position in the genome (red two-sided arrows).

To narrow down the causal sequence/region underlying the allelic effect of *RDP1*, we performed functional experiments using natural accessions. We first performed a traditional transgenic test to detect the allelic effect of *RDP1* (Figure 2A), but found that the resolution was not sufficient to detect it, as follows. The prepared *RDP1* sequences ranged from the upstream region (1,960 bp in Col-0) to the downstream region (661 bp in Col-0, Supplementary Figure 1) of three *A. thaliana* accessions (Col-0, Mz-0, and Bor-4), as well as *A. lyrata* ssp. *lyrata.* Mz-0 was among the accessions with the lowest pollen number, and the predominantly outcrossing species *A. lyrata* produced a much greater number of pollen grains than the *A. thaliana* accessions (Tsuchimatsu et al., 2020). Each sequence was introduced into the CRISPR/Cas9-generated *rdp1* null (non-functional) mutant (*rdp1-3/rdp1-3*, Col-0 background), and more than 10 independent transformants were obtained for each of the four constructs. However, the variation in pollen number was high, even among the transformants of the same construct, and the range of the number of pollen grains largely overlapped between the four constructs (Supplementary Figures 2A,B). When these data were compared with those obtained for wild-type Col-0, three of them showed no significant difference, whereas, unexpectedly, the construct with Col-0 showed a slight increase in pollen number, although a previous complementation test on another *rdp1* mutant background (*rdp1-1*) showed no significant difference compared with Col-0 (Tsuchimatsu et al., 2020). We speculate that the positional effect of the insertion site was too large to detect a subtle allelic difference. In *Arabidopsis* transgenic experiments, it is difficult to control the insertion sites of transgenes. Depending on the insertion position, the expression level, timing, and/or tissue type of the transgenes may be affected and other genes can be disrupted. Although a drastic increase in the number of independent transgenic lines may eventually result in significant differences, the construction of these lines and the measurement of phenotypes would be highly tedious.

**FIGURE 3:**
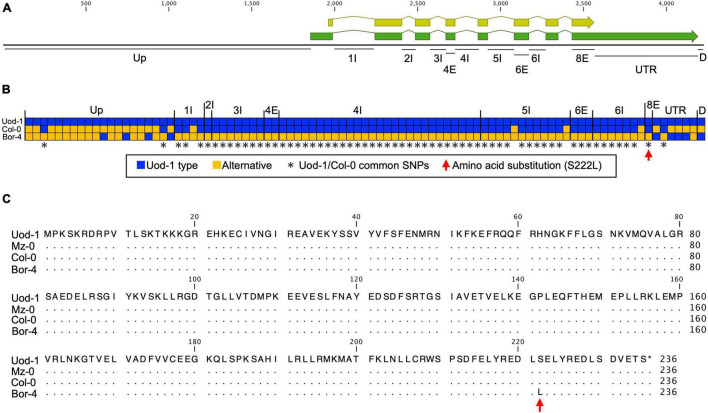
Comparison of *RDP1* sequences among Uod-1, Col-0, and Bor-4. **(A)** Schematic structure of the *RDP1* gene. The green and yellow boxes indicate mRNA and coding regions, respectively. Up, upstream; I, intron; E, exon; UTR, 3′UTR; D, downstream. Each annotation was defined by NCBI information (NM_102335.4). The number with I or E stands for the ordinal number of each intron or exon. Only the names of polymorphic regions are indicated. **(B)** Haplotype map of the *RDP1* gene. All SNPs and gaps (indels) of the *RDP1* region are shown. Each box corresponds to a polymorphism. Continuous gaps (indels) are displayed as a single box. The blue box indicates the Uod-1 type, and the orange box indicates an alternative polymorphism. The asterisks indicate common SNPs between Uod-1 and Col-0. The red arrow indicates an amino acid substitution in *RDP1* (S222L). The nucleic acid sequences of the *RDP1* region corresponding to **(A)** region from Uod-1, Col-0, and Bor-4 are shown in Supplementary Figure 1. **(C)** Amino acid sequences of *RDP1* from Uod-1, Mz-0, Col-0, and Bor-4. Dots represent identical sequence with Uod-1. Single amino acid substitution alone was identified among these accessions (S222L, red arrow). Sequence annotations and multiple sequence alignment were generated using the CLC Main Workbench (version 21.0.5)(**A,C**, and Supplementary Figure 1).

We then used the quantitative complementation test to detect the allelic effect of *RDP1*. Previously, we showed the allelic differences of *RDP1* between Uod-1 (a variant conferring reduced pollen number) and Bor-4 (a variant conferring increased pollen number) via the quantitative complementation test (Figure 2B; Tsuchimatsu et al., 2020). Here, we tested quantitative complementation using the Bor-4 and Col-0 accessions. We aligned the *RDP1* genomic region of Col-0 to that of Uod-1 and Bor-4 (Figure 3A and Supplementary Figure 1). The analysis of the alignment of genomic sequences showed that Col-0 experienced a historical recombination event near the start of the transcribed region, i.e., between its upstream and transcribed (exon/intron) regions (Figure 3B; Tsuchimatsu et al., 2020). The upstream sequence of Col-0 was close to that of Bor-4. However, the exonic and intronic sequences of Col-0 were very close to those of Uod-1, and there was only a single amino acid substitution differentiating Bor-4 from the three other accessions (S222L, Figures 3B,C). This amino acid position was deduced to be functionally important because the corresponding serine residues at the C-terminal region of human Mrt4 regulate cellular localization under the stress condition (Michalec-Wawiorka et al., 2015). In addition, the 3′UTR and downstream regions of Col-0 had several unique substitutions compared with Uod-1 and Bor-4. Phenotypically, Col-0 and Uod-1 had smaller numbers of pollen grains (Col-0, 3,355 pollen grains/flower; Uod-1, 3,277 pollen grains/flower) in contrast with Bor-4 (4,528 pollen grains/flower) (Tsuchimatsu et al., 2020). The unique sequence character of Col-0 prompted us to study whether the coding region has a variant conferring reduced pollen number.

**FIGURE 4:**
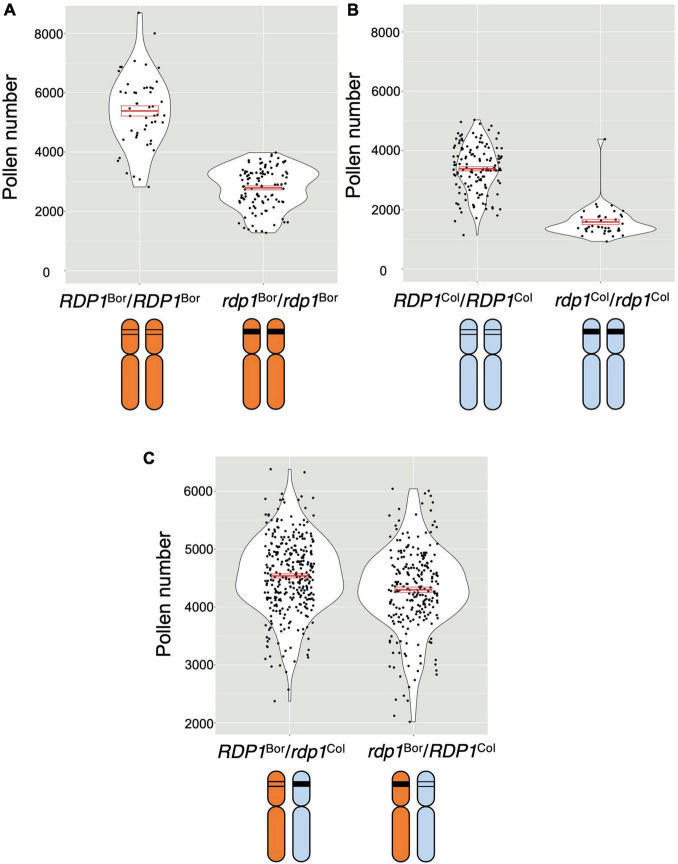
Quantitative complementation test of the *RDP1* gene. Pollen number per flower was analyzed. Violin plots with means and standard errors of means indicated by red bold bars and boxes, respectively. **(A,B)** Pollen-number difference between the wild-type and a homozygote of a frameshift allele generated by the CRISPR/Cas9 technique in the Bor-4 background (**A**; *n* = 50 [*RDP1*^Bor^*/RDP1*^Bor^]; *n* = 106 [*rdp1*^Bor^*/rdp1*^Bor^]) and in the Col-0 background (**B**; *n* = 135 [*RDP1*^Col^*/RDP1*^Col^]; *n* = 40 [*rdp1* ^Col^*/rdp1*^Col^]). (**C)** Difference in the effect of two natural alleles, *RDP1*^Bor^ and *RDP1*^Col^, on pollen number. The pollen number in the plants with *RDP1*^Bor^ was significantly higher than that in the plants with *RDP1*^Col^ (nested analysis of variance; *P* = 1.10 × 10^–5^; *n* = 315 [*RDP1*^Bor^*/rdp1*^Col^, median = 4,528 pollen grains/flower]; *n* = 247 [*rdp1*^Bor^*/RDP1*^Col^, median = 4,302 pollen grains/flower]). The two alleles were compared in the heterozygous state with a frameshift CRISPR/Cas9 allele in an identical genomic background. F_1_ plants were obtained from the cross of two heterozygotes, *RDP1*^Col^*/rdp1*^Col^ and *RDP1*^Bor^*/rdp1*^Bor^. Results from four genotypes including *RDP1*^Bor^*/RDP1*^Col^ and *rdp1*^Bor^*/rdp1*^Col^ were shown in Supplementary Figure 3.

To compare the *RDP1* allele, plants were prepared as follows (Figure 2B): (1) *RDP1* heterozygous plants (*RDP1/rdp1*) were prepared from the Bor-4 and Col-0 accessions; (2) two heterozygous plants were crossed; and (3) *RDP1*^Bor^/*rdp1*^Col^ and *rdp1*^Bor^/*RDP1*^Col^ plants were selected. These plants had a single different functional *RDP1* allele in the original chromosomal position, but all other genome sequences were identical: a single disrupted *RDP1* allele with the same frameshift position and the same genome constitution (one chromosome from Bor-4 and the other from Col-0) outside of *RDP1*. We found that the plants with the functional Bor-4 allele of *RDP1* (*RDP1*^Bor^/*rdp1*^Col^) had a significantly higher pollen number than those with a functional Col-0 allele (*rdp1*^Bor^/*RDP1*^Col^) (*P* = 1.10 × 10^–5^; Figure 4). We estimated allelic effect of *RDP1* based on median in the quantitative complementation test. *RDP1*^Bor^ is 1,599 pollen grain increasing [*RDP1*^Bor^/*rdp1*^Col^ (4,528 pollen grain) vs. *rdp1*^Bor^/*rdp1*^Col^ (2,929 pollen grain)] and *RDP1*^Col^ is 1,373 pollen grain increasing [*rdp1*^Bor^/*RDP1*^Col^ (4,302 pollen grain) vs. *rdp1*^Bor^/*rdp1*^Col^ (2,929 pollen grain)] (Supplementary Figure 3). This result elucidated the allelic difference between Bor-4 and Col-0.

### Enhanced Transcription of Ribosome-Related Genes and the Reduction of That of Specific Anther/Pollen Development Genes in *rdp1-3*

**FIGURE 5:**
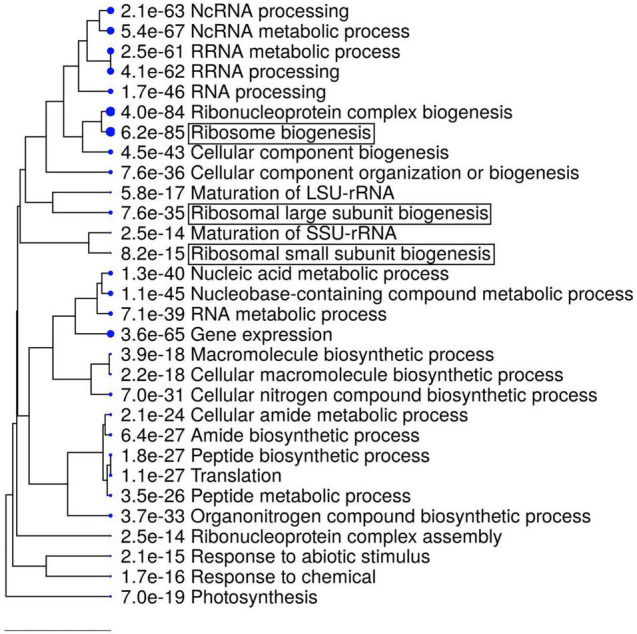
Gene ontology (GO) enrichment analysis of upregulated differentially expressed genes (DEGs) in *rdp1* compared with the wild-type (1284 genes). The top 30 hits on the GO list are shown in this figure, and the top 500 hits in the GO data are listed in Supplementary Table 3. Hierarchical clustering tree summarizing the correlation among the significant pathways listed in the Enrichment tab. Larger dots indicate smaller *P*-values. GO terms containing “ribosome” are highlighted by boxes.

Next, we examined which biological processes are disturbed by the disruption of the *RDP1* gene in flower bud tissues, and whether the expression of ribosome-related genes is altered as previously described for other *Arabidopsis* ribosome mutants. We performed a transcriptome analysis using wild-type of Col-0 and *rdp1-3* plants. A large number of DEGs between the wild-type and *rdp1-3* plants was detected using the criterion of FDR < 0.1 (3,020 genes, Supplementary Table 2). Among them, 1,284 genes were upregulated and 1,736 genes were downregulated in *rdp1-3* plants. A GO term analysis showed the enrichment of many ribosome-related genes in the upregulated gene set (Figure 5 and Table 1 and Supplementary Table 3). These results strongly support the notion that *RDP1* of *A. thaliana* functions in the biogenesis of the ribosome as a yeast Mrt4 homolog (Lo et al., 2010; Michalec-Wawiorka et al., 2015; Greber, 2016; Saez-Vasquez and Delseny, 2019).

**TABLE 1:** DEGs from ribosomal small and large subunit-related genes (FDR < 0.1).

Locus	RP family	RP name	WT_FPKM	*rdp1-3*_FPKM	Fold change (*rdp1-3*/WT)	Up/Down	FDR
**Small subunit**							
AT2G17360	S4	RPS4A	301.750	417.175	1.383	Up	0.03901
AT2G37270	S5	RPS5A	174.325	242.650	1.392	Up	0.04229
AT4G31700	S6	RPS6A	500.050	716.100	1.432	Up	0.03465
AT5G39850	S9	RPS9C	21.920	29.573	1.349	Up	0.08566
AT5G09500	S15	RPS15C	270.500	139.763	0.517	Down	0.0001105
AT5G63070	S15	RPS15F	2.147	0.656	0.306	Down	0.003703
AT3G04920	S24	RPS24A	362.125	472.675	1.305	Up	0.08819
AT1G23410	S27a	RPS27aA	20.613	33.018	1.602	Up	0.002746
**Large subunit**							
AT2G40010	P0	RPP0A	33.220	45.148	1.359	Up	0.0401
AT3G09200	P0	RPP0B	730.525	1018.125	1.394	Up	0.05074
AT1G01100	P1	RPP1A	172.625	249.500	1.445	Up	0.01443
AT5G47700	P1	RPP1C	225.475	291.725	1.294	Up	0.08017
AT4G25890	P3	RPP3A	49.863	70.398	1.412	Up	0.07064
AT1G61580	L3	RPL3B	6.506	4.296	0.660	Down	0.0688
AT3G09630	L4	RPL4A	219.625	353.675	1.610	Up	0.0004233
AT5G02870	L4	RPL4D	289.350	475.600	1.644	Up	0.001281
AT5G39740	L5	RPL5B	165.500	214.700	1.297	Up	0.08304
AT1G80750	L7	RPL7A	47.308	63.925	1.351	Up	0.04856
AT2G44120	L7	RPL7C	221.250	318.225	1.438	Up	0.02429
AT2G47610	L7a	RPL7aA	440.875	581.925	1.320	Up	0.08774
AT2G18020	L8	RPL8A	771.800	1106.525	1.434	Up	0.03117
AT4G36130	L8	RPL8C	64.268	89.533	1.393	Up	0.02703
AT4G10450	L9	RPL9D	39.513	65.113	1.648	Up	0.0004775
AT5G22440	L10a	RPL10aC	117.043	154.350	1.319	Up	0.09077
AT3G58700	L11	RPL11B	38.113	49.583	1.301	Up	0.09166
AT5G45775	L11	RPL11D	305.325	395.375	1.295	Up	0.09226
AT2G37190	L12	RPL12A	131.750	172.075	1.306	Up	0.07142
AT3G53430	L12	RPL12B	425.450	552.250	1.298	Up	0.0906
AT5G60670	L12	RPL12C	160.125	216.100	1.350	Up	0.08106
AT3G07110	L13a	RPL13aA—	368.900	574.000	1.556	Up	0.004389
AT2G20450	L14	RPL14A	54.750	78.120	1.427	Up	0.01233
AT4G17390	L15	RPL15B	462.025	610.425	1.321	Up	0.08518
AT3G16780	L19	RPL19B	46.078	68.470	1.486	Up	0.0467
AT2G33370	L23	RPL23B	120.215	164.650	1.370	Up	0.0819
AT2G39460	L23a	RPL23aA	1465.250	1984.000	1.354	Up	0.09487
AT2G44860	L24	RPL24C	108.735	145.025	1.334	Up	0.06166
AT3G49910	L26	RPL26A	496.375	711.675	1.434	Up	0.04323
AT4G29410	L28	RPL28C	120.538	178.900	1.484	Up	0.007889
AT4G18100	L32	RPL32A	332.650	442.025	1.329	Up	0.06263
AT3G28900	L34	RPL34C	149.325	193.750	1.298	Up	0.06931
AT2G39390	L35	RPL35B	22.165	32.263	1.456	Up	0.06232

*The pink and blue highlights indicate gene upregulation and downregulation, respectively. The list of cytosolic ribosomal genes was taken from previous reports (Barakat et al., 2001; Hummel et al., 2015).*

**FIGURE 6:**
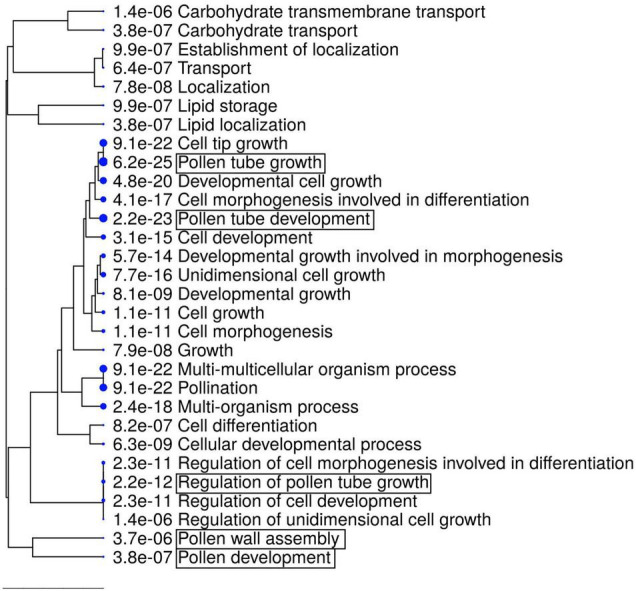
GO enrichment analysis of downregulated DEGs (1736 genes). The top 30 hits on the GO list are shown in this figure, and all GO data are listed in Supplementary Table 4. Hierarchical clustering tree summarizing the correlation among the significant pathways listed in the Enrichment tab. Larger dots indicate smaller *P*-values. GO terms containing “pollen” are highlighted by boxes.

The GO term analysis also revealed the enrichment of genes relevant for pollen development, as well as pollen-related categories in the downregulated gene set (e.g., “Pollen development,” “Pollen wall assembly,” and “Pollen tube growth”; Figure 6 and Supplementary Table 4). Among the genes relevant for pollen development, three bHLH transcription factor genes, *bHLH010*, *bHLH089*, and *AMS*, were downregulated (Table 2), all of which interact with the DYT1 during anther development (Feng et al., 2012; Ferguson et al., 2017). Although their fold change itself was modest (29.7–34.4% reduction; Table 2), we also observed a systematic downregulation of genes that were directly regulated by AMS (19 out of 23, *P* = 2.901e–15, Fisher’s exact test; Table 2; Xu et al., 2014). These data suggest that the bHLH pathway and its downstream genes were downregulated in the *rdp1-3* mutant. By contrast, we did not find any enrichment of GO terms related to ovule development, although ovule number was also reduced in the *rdp1-3* (Tsuchimatsu et al., 2020). This expression analysis in the flower bud suggests a distinctive role for *RDP1* in anther development through transcription factor regulation.

**TABLE 2:** Downregulated pollen/anther development-related genes.

ID	Gene symbol	Gene name/description	FPKM		Fold change	Up/Down	FDR
						
			WT	*rdp1-3*	(*rdp1*-*3*/WT)		
**Fifty-one downregulated genes in GO “Pollen development” (51 of 344 genes)**							
AT4G27420	ABCG9	ATP-BINDING CASSETTE G9	10.36	6.73675	0.650	Down	0.03768
AT3G13220	ABCG26	ATP-BINDING CASSETTE G26	32.95	24.17	0.734	Down	0.07096
AT3G21180	ACA9	AUTOINHIBITED CA(2 +)-ATPASE 9	13.565	5.79125	0.427	Down	0.001642
AT2G03060	AGL30	AGAMOUS-LIKE 30	20.29	11.8888	0.586	Down	0.003651
AT1G77980	AGL66	AGAMOUS-LIKE 66	9.15675	3.65205	0.399	Down	0.01597
AT1G69540	AGL94	AGAMOUS-LIKE 94	4.50075	2.462	0.547	Down	0.01472
AT1G22130	AGL104	AGAMOUS-LIKE 104	21.21	10.585	0.499	Down	0.006843
AT5G14380	AGP6	ARABINOGALACTAN PROTEIN 6	695.075	212.25	0.305	Down	0.0004339
AT2G16910	AMS	ABORTED MICROSPORES	20.7625	13.3375	0.642	Down	0.009175
AT5G54470	BBX29	B-BOX DOMAIN PROTEIN 29	1.01833	0.44235	0.434	Down	0.05074
AT2G31220	bHLH010	BASIC HELIX LOOP HELIX PROTEIN 10	11.089	7.27475	0.656	Down	0.03039
AT1G06170	bHLH089	BASIC HELIX LOOP HELIX PROTEIN 89	27.22	19.1275	0.703	Down	0.08885
AT3G57370	BRP4	transcription factor IIB-related protein	7.012	3.73475	0.533	Down	0.004574
AT5G59030	COPT1	COPPER TRANSPORTER 1	126.868	77.21	0.609	Down	0.03737
AT1G01280	CYP703A2	CYTOCHROME P450, FAMILY 703, SUBFAMILY A, POLYPEPTIDE 2	26.2025	18.22	0.695	Down	0.06944
AT3G04620	DAN1	D NUCLDUO1-ACTIVATEEIC ACID BINDING PROTEIN 1	50.7075	29.4975	0.582	Down	0.002855
AT3G60460	DUO1	DUO POLLEN 1	2.4205	1.26045	0.521	Down	0.07064
AT4G10950	GELP77	GDSL-TYPE ESTERASE/LIPASE 77	2.621	1.49225	0.569	Down	0.07858
AT5G54800	GPT1	GLUCOSE 6-PHOSPHATE/PHOSPHATE TRANSLOCATOR 1	55.88	36.22	0.648	Down	0.02275
AT4G22600	INP1	INAPERTURATE POLLEN1	1.95575	0.9254	0.473	Down	0.06207
AT1G19180	JAZ1	JASMONATE-ZIM-DOMAIN PROTEIN 1	36.1125	22.34	0.619	Down	0.01786
AT5G11110	KNS2	KAONASHI 2	6.718	4.33975	0.646	Down	0.02011
AT2G35210	MEE28	MATERNAL EFFECT EMBRYO ARREST 28	5.495	2.2425	0.408	Down	0.01798
AT4G14080	MEE48	MATERNAL EFFECT EMBRYO ARREST 48	445.275	249.95	0.561	Down	0.00152
AT1G19890	MGH3	MALE-GAMETE-SPECIFIC HISTONE H3	5.827	3.23475	0.555	Down	0.06423
AT3G11980	MS2	MALE STERILITY 2	85.015	59.585	0.701	Down	0.05185
AT5G46795	MSP2	MICROSPORE-SPECIFIC PROMOTER 2	28.515	16.8475	0.591	Down	0.004003
AT2G26960	MYB81	MYB DOMAIN PROTEIN 81	9.4635	6.213	0.657	Down	0.06235
AT2G32460	MYB101	MYB DOMAIN PROTEIN 101	22.33	14.6225	0.655	Down	0.06215
AT5G41090	NAC095	NAC DOMAIN CONTAINING PROTEIN 95	8.23725	4.3635	0.530	Down	0.02754
AT2G29940	PDR3	PLEIOTROPIC DRUG RESISTANCE 3	8.95675	5.879	0.656	Down	0.02452
AT5G05850	PIRL1	PLANT INTRACELLULAR RAS GROUP-RELATED LRR 1	19.8625	11.5435	0.581	Down	0.02079
AT4G34850	PKSB	POLYKETIDE SYNTHASE B	95.7175	67.7175	0.707	Down	0.09108
AT4G29470	PLA2-DELTA	PHOSPHOLIPASE A2 DELTA	5.387	1.73778	0.323	Down	0.005243
AT4G29460	PLA2-GAMMA	PHOSPHOLIPASE A2 GAMMA	0.65675	0.07614	0.116	Down	0.004069
AT5G39400	PTEN1	PHOSPHATASE AND TENSIN HOMOLOG DELETED ON CHROMOSOME TEN 1	3.12565	0.68463	0.219	Down	0.09549
AT4G20050	QRT3	QUARTET 3	68.4625	43.4175	0.634	Down	0.004464
AT4G17530	RAB1C	RAB GTPASE HOMOLOG 1C	70.8125	46.1975	0.652	Down	0.01514
AT5G40260	RPG1	RUPTURED POLLEN GRAIN1	12.3375	7.75825	0.629	Down	0.04411
AT5G50800	RPG2	RUPTURED POLLEN GRAIN 2	19.1675	6.2265	0.325	Down	0.0006463
AT1G09180	SAR1	SECRETION-ASSOCIATED RAS 1	4.53925	2.48025	0.546	Down	0.06193
AT1G06515	ssSPTa	SMALL SUBUNIT OF SPT A	68.015	44.575	0.655	Down	0.03059
AT1G07340	STP2	SUGAR TRANSPORTER 2	39.175	27.415	0.700	Down	0.02665
AT3G47440	TIP5;1	TONOPLAST INTRINSIC PROTEIN 5;1	3.16325	1.8435	0.583	Down	0.09475
AT4G35420	TKPR1	TETRAKETIDE ALPHA-PYRONE REDUCTASE 1	265	163.175	0.616	Down	0.009035
AT1G68540	TKPR2	TETRAKETIDE ALPHA-PYRONE REDUCTASE 2	18.45	12.1625	0.659	Down	0.05092
AT4G12920	UND	UNDEAD	11.3435	6.2965	0.555	Down	0.04017
AT4G26440	WRKY34	WRKY DNA-BINDING PROTEIN 34	7.033	4.24575	0.604	Down	0.009744
AT2G35070		transmembrane protein;(source:Araport11)	8.2975	4.489	0.541	Down	0.01696
AT3G10470		C2H2-type zinc finger family protein;(source:Araport11)	1.9	0.54983	0.289	Down	0.06933
AT3G28780		transmembrane protein, putative (DUF1216);(source:Araport11)	127.82	40.83	0.319	Down	0.00002049
**Twenty-three genes directly regulated by AMS (Xu et al., 2014)**							
AT4G14080	MEE48	MATERNAL EFFECT EMBRYO ARREST 48	445.28	249.95	0.561	Down	0.00152
AT4G20050	QRT3	QUARTET 3	68.46	43.42	0.634	Down	0.004464
AT3G52160	KCS15	3-KETOACYL-COA SYNTHASE 15	47.29	35.54	0.752	n.s.	0.1359
AT5G49070	KCS21	03-KETOACYL-COA SYNTHASE 21	8.12	8.72	1.074	n.s.	0.8009
AT1G71160	KCS7	03-KETOACYL-COA SYNTHASE 7	14.41	12.24	0.850	n.s.	0.4758
AT3G51590	LTP12	LIPID TRANSFER PROTEIN 12	2847.00	1431.10	0.503	Down	0.00206
AT1G66850		LTP family protein	1532.75	815.58	0.532	Down	0.00003572
AT5G62080		LTP family protein	519.88	285.28	0.549	Down	0.001196
AT3G13220	ABCG26	ATP-Binding Cassette G subfamily 26	32.95	24.17	0.734	Down	0.07096
AT4G34850	PKSB/LAP5	POLYKETIDE SYNTHASE B/LESS ADHESIVE POLLEN 5	95.72	67.72	0.707	Down	0.09108
AT4G35420	TKPR1	TETRAKETIDE ALPHA-PYRONE REDUCTASE 1	265.00	163.18	0.616	Down	0.009035
AT4G00040	CHS	Chalcone and stilbene synthase family protein	82.51	55.72	0.675	Down	0.01389
AT1G75920	EXL5	EXTRACELLULAR LIPASE 5	54.14	27.89	0.515	Down	0.000005513
AT1G75910	EXL4	EXTRACELLULAR LIPASE 4	408.30	210.20	0.515	Down	0.000004671
AT1G75930	EXL6	EXTRACELLULAR LIPASE 6	244.13	120.38	0.493	Down	7.601e–07
AT1G06990		GDSL-like Lipase/Acylhydrolase superfamily protein	32.87	19.16	0.583	Down	0.003785
AT1G69500	CYP704B1	CYTOCHROME P450, FAMILY 704, SUBFAMILY B, POLYPEPTIDE 1	14.82	11.20	0.756	n.s.	0.219
AT1G01280	CYP703A2	CYTOCHROME P450, FAMILY 703, SUBFAMILY A, POLYPEPTIDE 2	26.20	18.22	0.695	Down	0.06944
AT1G74540	CYP98A8	CYTOCHROME P450, FAMILY 98, SUBFAMILY A, POLYPEPTIDE 8	40.58	27.55	0.679	Down	0.02009
AT1G74550	CYP98A9	CYTOCHROME P450, FAMILY 98, SUBFAMILY A, POLYPEPTIDE 9	55.92	37.84	0.677	Down	0.01419
AT1G13140	CYP86C3	CYTOCHROME P450, FAMILY 86, SUBFAMILY C, POLYPEPTIDE 3	59.11	43.66	0.739	Down	0.0959
AT5G07520	GRP18	GLYCINE-RICH PROTEIN 18	63.58	31.91	0.502	Down	1.898e–07
AT5G07550	GRP19	GLYCINE-RICH PROTEIN 19	5613.75	2498.00	0.445	Down	0.0002736

*The genes highlighted in blue are downregulated.*

## Discussion

### Quantitative Complementation Tests of Multiple Combinations to Narrow Down the Genomic Regions Responsible for Pollen-Number Regulation

Although the quantitative complementation test has been validated in several animal and bacterial species, such as yeast, *Drosophila*, mosquito, fish, and mouse (Steinmetz et al., 2002; Stern, 2014; Turner, 2014; Podobnik et al., 2020), the preparation of mutants in a specific site of a gene represents a bottleneck regarding the application of quantitative complementation tests in plants (Stern, 2014). Recent progress in genome-editing technologies has allowed the efficient generation of frameshift non-functional mutants at the targeted gene in several different accessions. Thus, a quantitative complementation test will have a broader application of obtaining causal evidence of subtle allelic effects in plant species.

In this study, we performed a quantitative complementation test of *RDP1* between Col-0 and Bor-4. A key of the method is to generate equivalent non-functional alleles in two different backgrounds by CRISPR/Cas9. We generated frameshift mutants at the same position, i.e., *rdp1-3* and *rdp1-6* in Col-0 and Bor-4, respectively. We consider them as null mutants because frameshift mutants are in general disruptive, and the yeast studies suggested the importance of C-terminal domains (Michalec et al., 2010), but we note that the quantitative complementation test can work even if they are not completely null. Combined with the previously reported results of the quantitative complementation test between Uod-1 and Bor-4 (Tsuchimatsu et al., 2020), our data support the notion that both Col-0 and Uod-1 have alleles that confer reduced pollen number against Bor-4. Although it is formally possible that more than two functional variants exist, the selective sweep signature of the long-haplotype variants suggests that a single mutation conferring reduced pollen number spread to many accessions, including Col-0 and Uod-1. This implies that the causal mutation of the reduced pollen number is shared between Col-0 and Uod-1, in contrast with Bor-4. The alignment of the genomic sequences of the three accessions revealed that such substitutions were mostly found in exon/intron regions, and not in upstream or downstream regions, reflecting the historical recombination inferred in Col-0. Although it is possible that mutations outside of the aligned region may have contributed to this phenomenon, substitutions in the exon/intron regions, including S222L, are promising candidate causal mutations of the reduced pollen number (Figures 3B,C), which warrants further experimental verification. In short, quantitative complementation tests using multiple accessions serve as a tool to narrow down the candidate regions of causal mutations of quantitative traits when transgenic experiments are not sufficiently powerful.

### *RDP1* and the Biogenesis of the Ribosomal Large Subunit

Remarkably, we detected two ontologies related to the ribosomal large subunit (“Ribosomal large subunit assembly” and “Ribosomal large subunit biosynthesis”) in the transcriptome analysis of *rdp1-3*. We found that 23.0% of the genes involved in the ribosomal large subunit were significantly differentially expressed (33 out of 143 genes), with most of them being upregulated (32 out of 33 genes) (Table 1). Conversely, only 8.2% of the genes involved in the ribosomal small subunit were significantly different (8 out of 98 genes). Furthermore, S222L was the only non-synonymous mutation in the mapped region revealed by the two complementation tests; thus, it was a candidate causal mutation for the pollen-number phenotype (as discussed above). Moreover, this amino acid position was shown to be important for the cellular localization and ribosomal function of human *RDP1* (Michalec-Wawiorka et al., 2015). Taken together, our results strongly support the contention that *RDP1* of *A. thaliana* functions in the biogenesis of the ribosomal large subunit as a yeast Mrt4 homolog (Lo et al., 2010; Michalec-Wawiorka et al., 2015; Greber, 2016; Saez-Vasquez and Delseny, 2019).

### Regulation of the Expression of Pollen Development Genes in the Mutant of *RDP1*

The mechanisms underlying where and how *RDP1* affects the expression of bHLH transcriptional factor genes, such as *AMS*, *bHLH010*, and *bHLH089*, remain unknown. Previous studies have reported that the spatial expression pattern of these transcriptional factor genes and that of *RDP1* overlap partly in the same cell types (Zhu et al., 2011; Zhu et al., 2015; Tsuchimatsu et al., 2020). Real-time PCR, promoter-GUS assay, and *in situ* hybridization studies have suggested that *RDP1* is expressed broadly and strongly in proliferating cells, including sporogenous cells (Tsuchimatsu et al., 2020). A transcriptome study of dissected tissues also demonstrated that *RDP1* is expressed in both tapetal cells and the pollen lineage (microspore, bicellular, and tricellular stages) (Honys and Twell, 2004; Li et al., 2017). The expressions of *bHLH010* and *bHLH089* were detected in both microspores and tapetum cells via *in situ* hybridization (Zhu et al., 2015). Moreover, *in situ* hybridization suggested that *AMS* is expressed in both tapetum cells and microspores (Zhu et al., 2011), although the defect of *ams* mutants is primarily found in surrounding tapetum cells that provide pollen wall to the germ lines (Xu et al., 2010). These expression data suggest that these bHLH genes are co-expressed with *RDP1* in tapetum and microspore cells. It is reported that the feedback regulation of bHLH transcription factor genes (*DYT1*, *bHLH010*, *bHLH089*, and *bHLH091*) occurs during anther development (Cui et al., 2016). Because this feedback regulation requires the synthesis of the protein encoded by each gene, *RDP1* may contribute to the expression of these genes through translation by ribosomes. This hypothesis was supported by the observation that two of these genes (*bHLH010* and *bHLH089*) were downregulated in the *rdp1-3* mutant. We also considered the possibility that the transcriptional reduction of these bHLH genes in tapetal cells has an indirect effect on tapetum development via the microspore reduction afforded by *RDP1* mutation. However, this is unlikely because the expression patterns of other genes that are essential for tapetum development, such as *DYT1*, *TPD1*, and *MS188*, were not affected in *rdp1-3*. We could not exclude the possibility that the aberrant translational machinery of *rdp1-3* may affect the mRNA abundance, or of downstream transcription independent from the feedback (Tiruneh et al., 2013; Beine-Golovchuk et al., 2018; Cheong et al., 2021) or multi-functionalization of *RDP1* independent from the ribosome. Further detailed analyses are required to evaluate this scenario. In addition, previous transcriptome data obtained for the *bhlh* triple mutant (*bhlh010/bhlh089/bhlh091*) (Zhu et al., 2015) did not show an obvious transcriptional difference for ribosomal genes, including *RDP1*, suggesting that *RDP1* or ribosome function is not located downstream of the bHLH gene regulatory network.

### The Reduction in Pollen Number and Slower Growth Observed in the *rdp1-3* Mutant Are Possibly Caused by Ribosomal Specialization

The ribosome is a protein biosynthesis machinery that is present in all living cells. Although the ribosome has been considered a solid component common to all cells, organ-specific phenotypes are revealed from mutant analysis of ribosome and ribosome-biogenesis genes in yeast, humans, and plants. This ribosome specialization is now suggested as not being a rare phenomenon (Martinez-Seidel et al., 2020; Norris et al., 2021). Reproductive defects of ribosomal mutants were observed, such as reduced stamen number, smaller pollen size, slower pollen tube elongation, and reduced functional ovules (Stirnberg et al., 2012; Luo et al., 2020). *RDP1* is considered a homolog of yeast Mrt4, which is a key component for the 60S ribosomal assembly. We found that the expression level of *RDP1* varied among different cell types and was higher in proliferating cells (Tsuchimatsu et al., 2020). Natural variants under selection supported the critical role of *RDP1* in adaptive evolution (Tsuchimatsu et al., 2020). Furthermore, a non-functional *RDP1* mutant exhibited reduced pollen number and slower growth speed. These data support the idea that the high expression level of *RDP1* is required to respond to the high demand of ribosome production in proliferation cells. Interestingly, Tsuchimatsu et al. (2020) reported that quantitative complementation test with Uod-1 and Bor-1 showed allelic differences for pollen number but not growth speed (rosette size and bolting/flowering date). This result may suggest the ribosome specialization for pollen number. At first glance, this may be similar to cytoplasmic sterility, where the reduction of mitochondrial function results in pollen-specific phenotype, where high energy consumption may be necessary (Warmke and Lee, 1978; Chase, 2007). However, our transcriptome data does not support this idea because there was no GO term “mitochondria” in down-regulated genes. Further analysis of *RDP1* will provide clues regarding how ribosome specialization affects the pollen number regulation.

## Conclusion

A previous study showed that natural variation of the *RDP1* gene was responsible for pollen number in *A. thaliana*. Here, we showed that the standard accession Col-0 had a natural variant that reduced pollen number. Moreover, the frameshift mutant *rdp1*-3 in the Col-0 background confirmed pleiotropic effects, including that on flowering time. A transcriptome analysis of the *rdp1-3* mutant suggested the function of *RDP1* in ribosome assembly and pollen development. These data support the specialized function of the ribosome in pollen development.

## Data Availability Statement

The datasets presented in this study can be found in online repositories. The names of the repository/repositories and accession number(s) can be found in the Supplementary Table 1.

## Author Contributions

HK, TT, MY, and KKS planned and designed the study and wrote the manuscript. HK and MY performed the experiments. HK and TT analyzed the data and drew the figures. MH managed the sequencing data. All authors contributed to the article and approved the submitted version.

## Conflict of Interest

The authors declare that the research was conducted in the absence of any commercial or financial relationships that could be construed as a potential conflict of interest.

## Publisher’s Note

All claims expressed in this article are solely those of the authors and do not necessarily represent those of their affiliated organizations, or those of the publisher, the editors and the reviewers. Any product that may be evaluated in this article, or claim that may be made by its manufacturer, is not guaranteed or endorsed by the publisher.

## Supplementary Material

Supplementary Figure 1 Nucleic acid comparison of the *RDP1* region from Uod-1, Col-0, and Bor-4. The green and yellow boxes indicate mRNA and coding regions, respectively. Each annotation was defined by NCBI information (NM_102335.4).

Supplementary Figure 2 Traditional transgenic complementation test of pollen number per flower in the *rdp1-3* background. No significant difference was detected in each case in line-wise (**A)** and in allele-wise (**B)** analyses (*n* = 269 *(RDP1/RDP1)*, 908 *(rdp1-3/rdp1-3 + RDP1*^Col−0^), 932 *(rdp1-3/rdp1-3 + RDP1*^*Mz*−0^), 713 *(rdp1-3/rdp1-3 + RDP1*^Bor−4^), and 788 *(rdp1-3/rdp1-3 + RDP1*^*Alyrata*^)). Boxplots show center line: median; box limits: upper and lower quartiles; dots: outliers.

Supplementary Figure 3 Pollen number from four genotypes of quantitative complementation test between Bor-4 and Col-0 accession. Sample numbers are shown above the plots. Boxplots show center line: median; box limits: upper and lower quartiles; whiskers: not greater than 1.5 times the interquartile range; point: outlier. Violin shape corresponds to the density of data.

Supplementary Table 1 Sample information and read numbers of transcriptome analysis in this study.

Supplementary Table 2 List of differentially expressed genes (3020 genes) between wild-type and *rdp1-3* plants.

Supplementary Table 3 TOP500 GO enrichment list (Biological process) for upregulated DEGs in *rdp1-3* plants.

Supplementary Table 4 Complete GO enrichment list (Biological process) for downregulated DEGs in *rdp1-3* plants.

Supplementary Video 1 Time-lapse movie of *RDP1/RDP1* (left) and *rdp1-3/rdp1-3* (right) plants. Photos were taken every hour from 10h00 to 19h00 for 39 days under the long-day condition.

## Abbreviations

AMS: *ABORTED MICROSPORES*
bHLH: basic helix-loop-helix
DEG: differentially expressed gene
DYT1: *DYSFUNCTIONAL TAPETUM1*
FAA: formaldehyde acetic acid
FDR: false discovery rate
GO: gene ontology
Mrt4: mRNA turnover4
RDP1: *REDUCED POLLEN NUMBER1*.
