# Supplementary figures and images for: Pollen Number and Ribosome Gene Expression Altered in a Genome-Editing Mutant of REDUCED POLLEN NUMBER1 Gene
Source: Front Plant Sci. 2022 Jan 11;12:768584. doi: 10.3389/fpls.2021.768584 (PMC8787260; doi:10.3389/fpls.2021.768584)

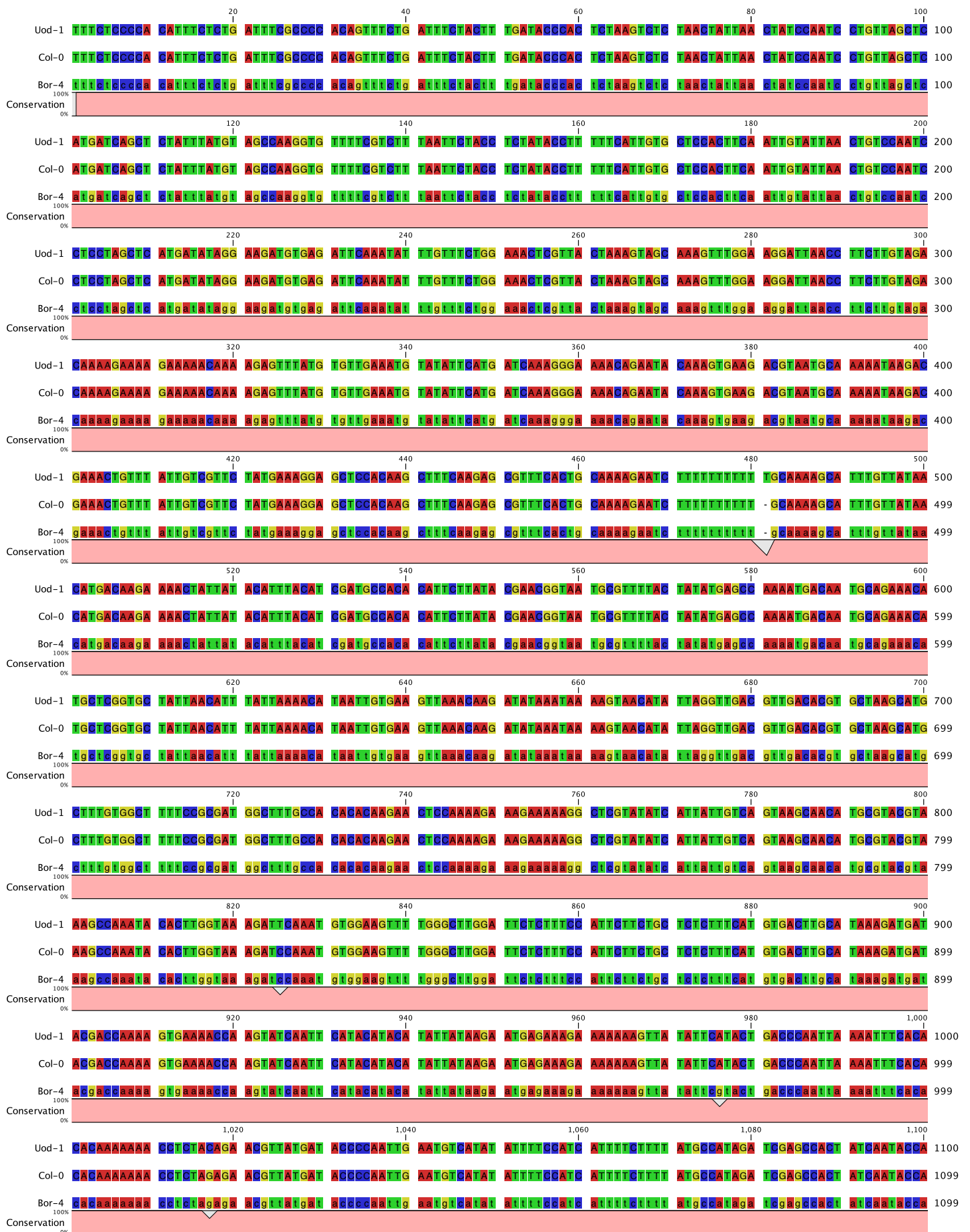

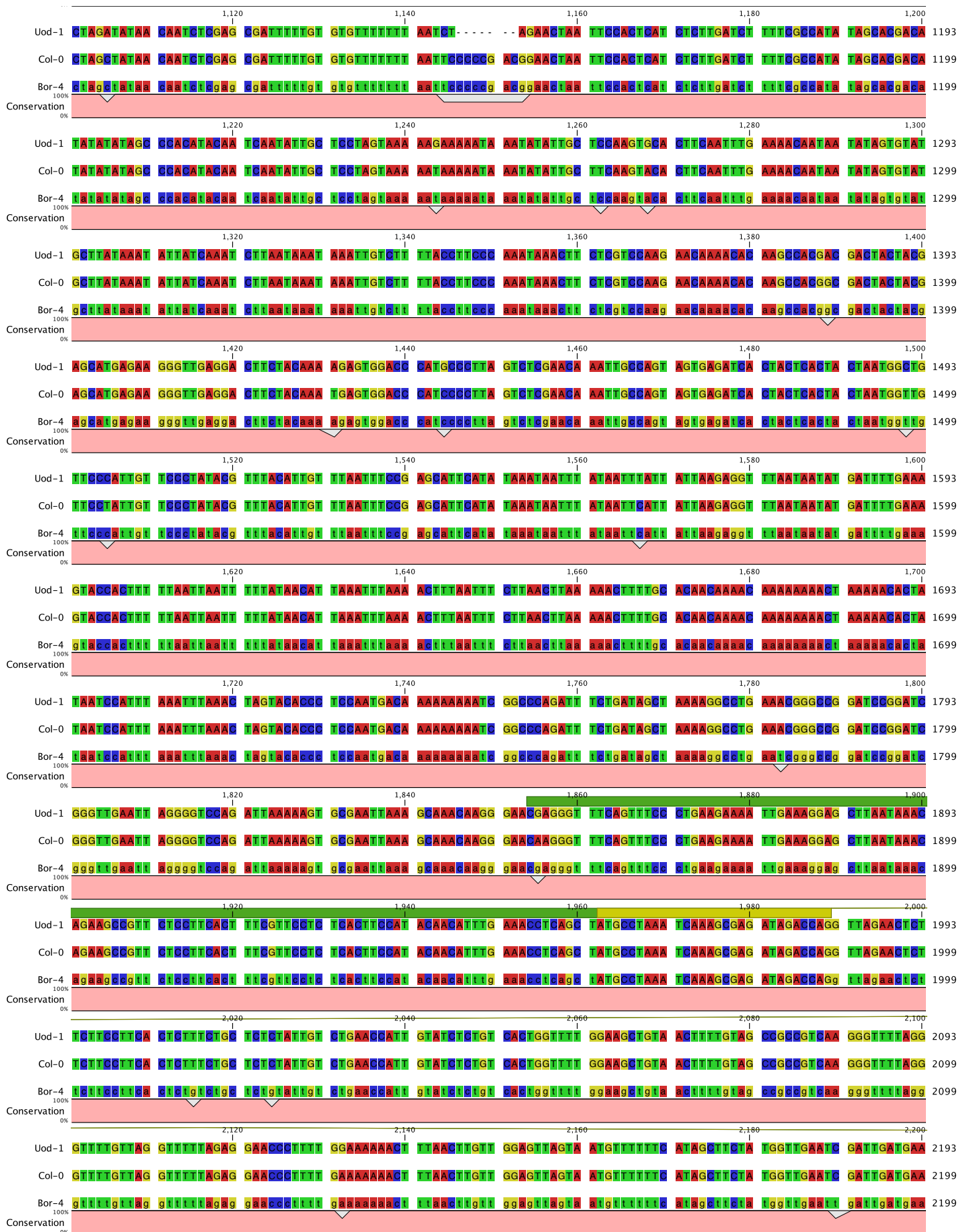

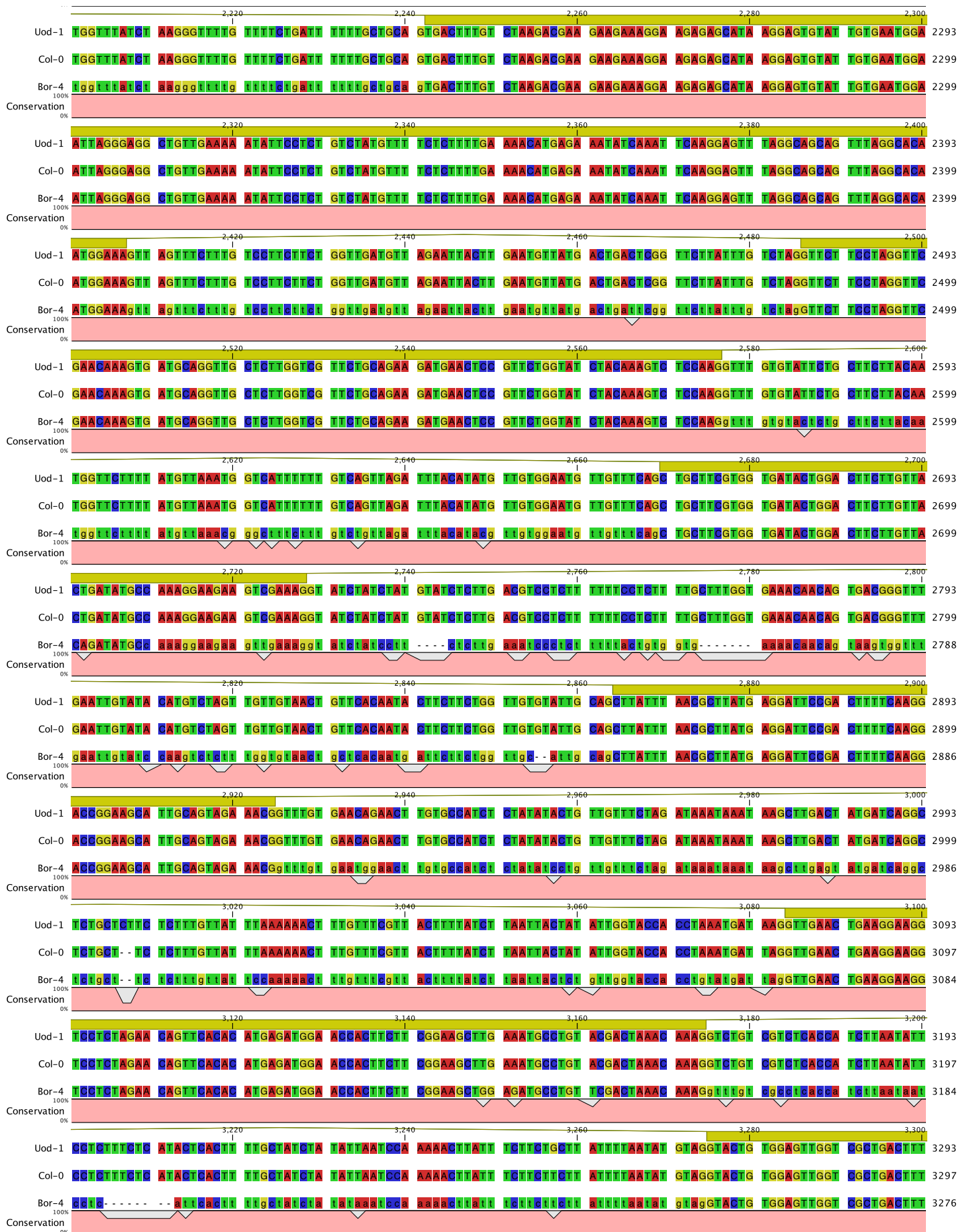

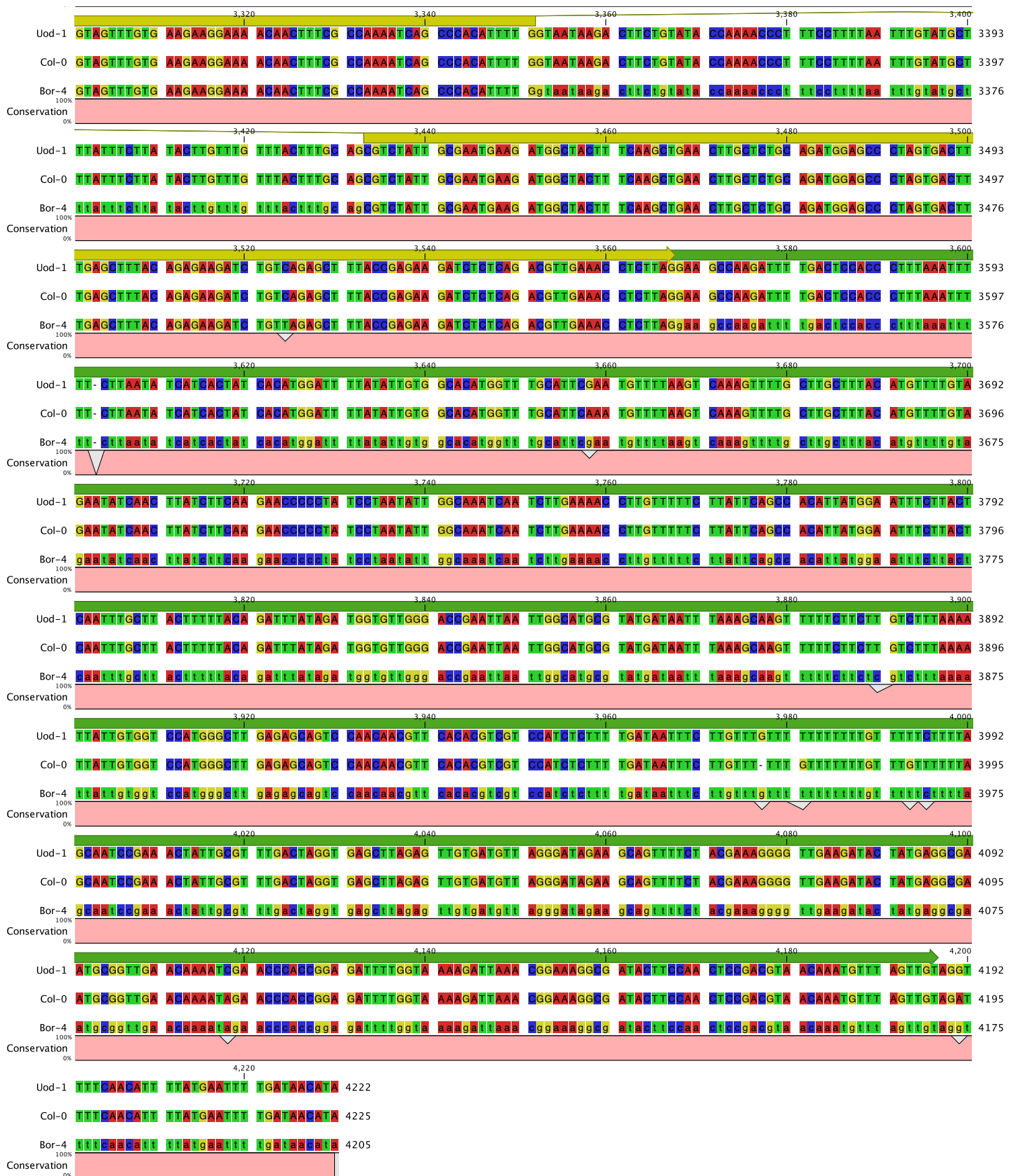

Supplement: Supplementary Figure 1 — Nucleic acid comparison of the RDP1 region from Uod-1, Col-0, and Bor-4. The green and yellow boxes indicate mRNA and coding regions, respectively. Each annotation was defined by NCBI information (NM_102335.4). [file Image_1.pdf]

A

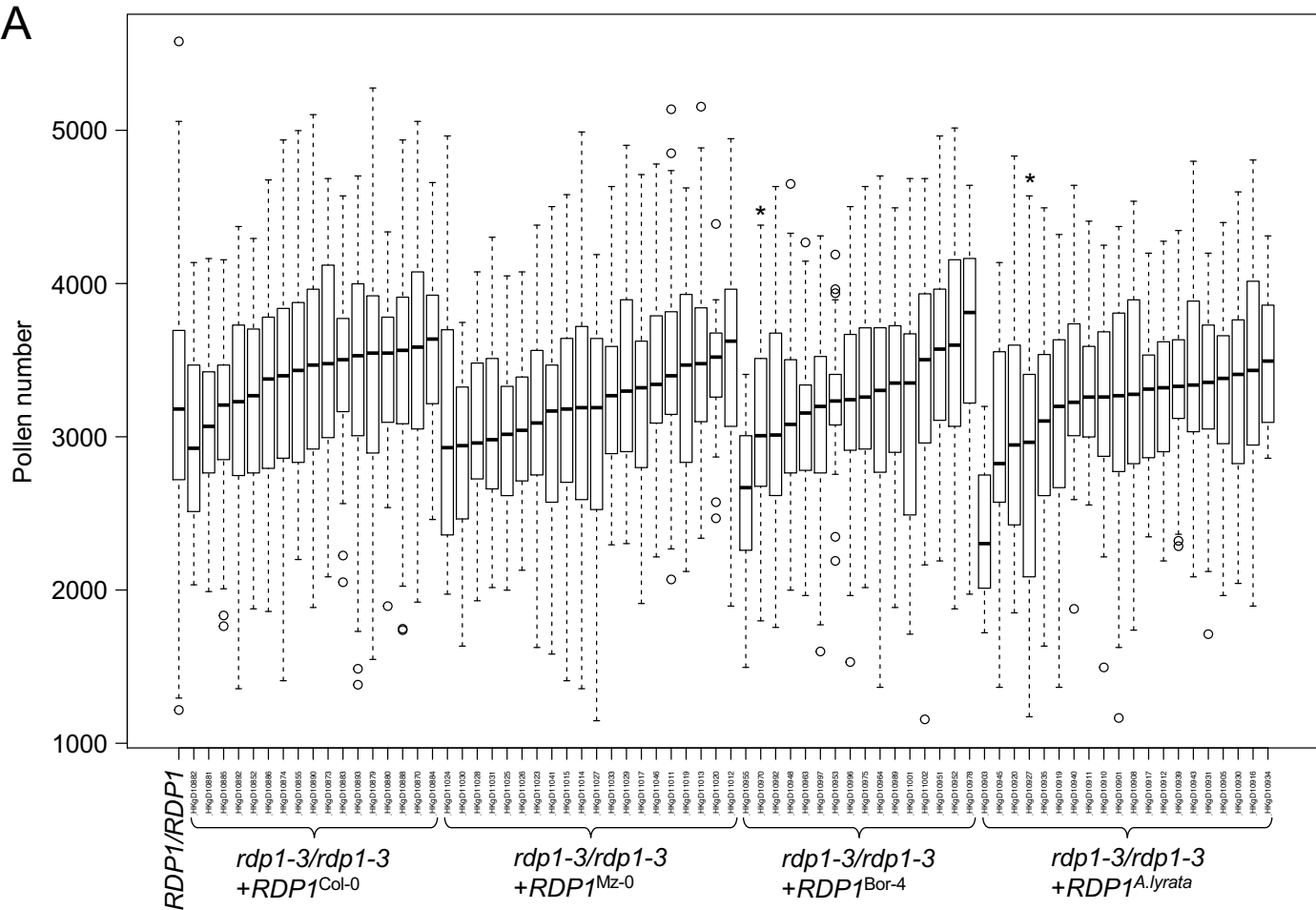

B

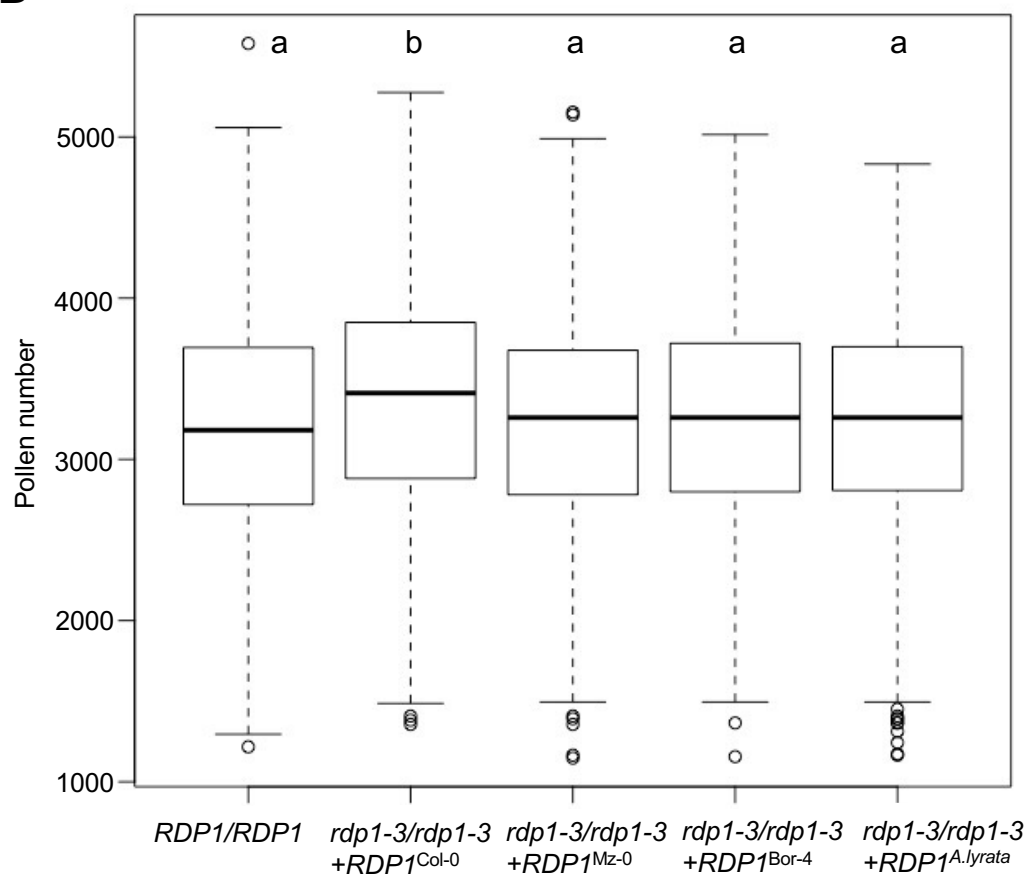

Supplement: Supplementary Figure 2 — Traditional transgenic complementation test of pollen number per flower in the rdp1-3 background. No significant difference was detected in each case in line-wise (A) and in allele-wise (B) analyses (n = 269 (RDP1/RDP1), 908 (rdp1-3/rdp1-3 + RDP1Col−0), 932 (rdp1-3/rdp1-3 + RDP1Mz−0), 713 (rdp1-3/rdp1-3 + RDP1Bor−4), and 788 (rdp1-3/rdp1-3 + RDP1Alyrata)). Boxplots show center line: median; box limits: upper and lower quartiles; dots: outliers. [file Image_2.pdf]

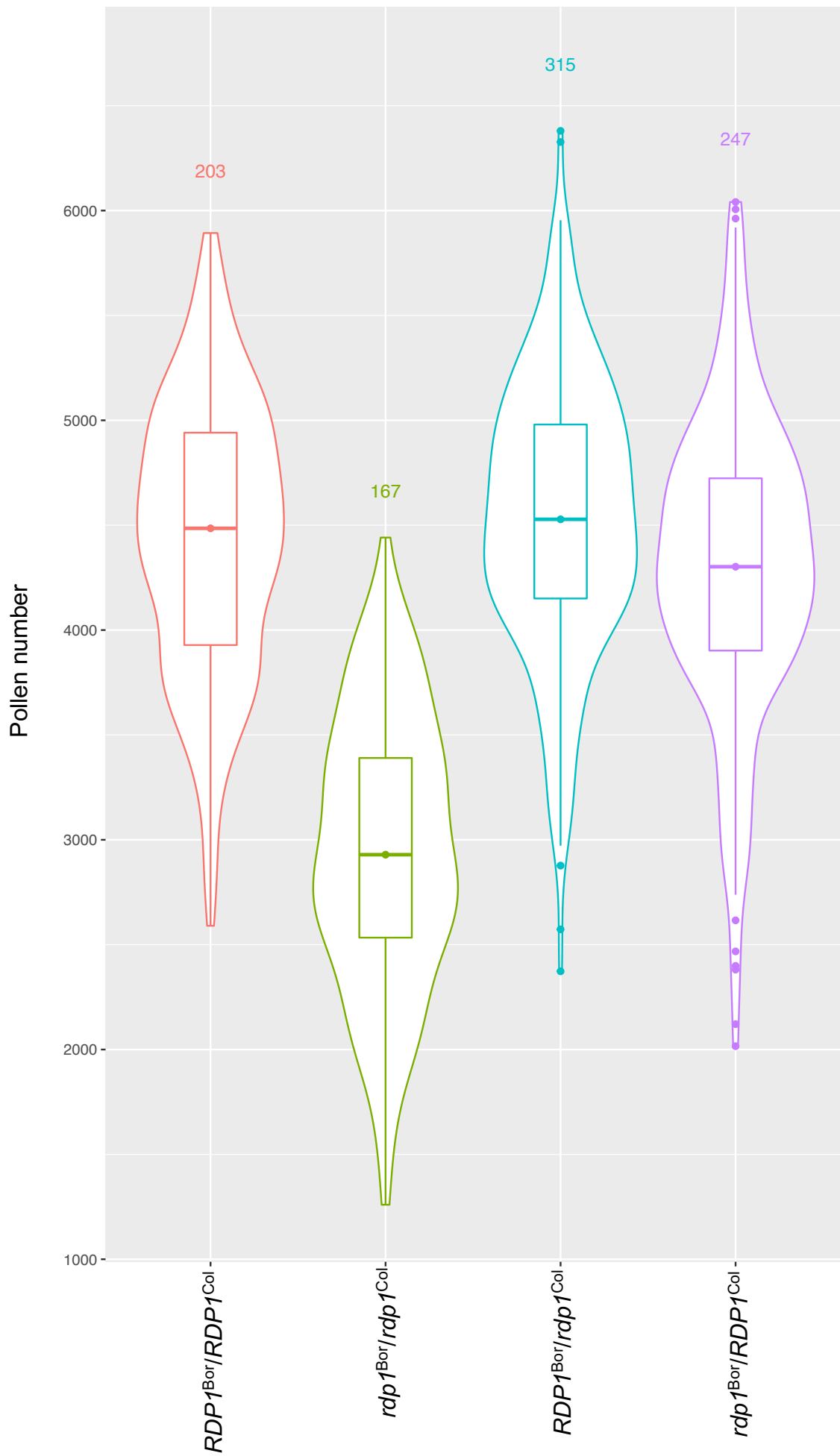

Supplement: Supplementary Figure 3 — Pollen number from four genotypes of quantitative complementation test between Bor-4 and Col-0 accession. Sample numbers are shown above the plots. Boxplots show center line: median; box limits: upper and lower quartiles; whiskers: not greater than 1.5 times the interquartile range; point: outlier. Violin shape corresponds to the density of data. [file Image_3.pdf]
